# Incidence of Recurrent Adverse Cardiovascular Events Among Patients With Acute Myocardial Infarction During the First Wave of the COVID‐19 Pandemic in Bangladesh: A Prospective Observational Study

**DOI:** 10.1002/hsr2.71254

**Published:** 2025-09-22

**Authors:** Zubair Akhtar, Fahmida Chowdhury, Mohammad Abdul Aleem, Mahmudur Rahman, Mustafizur Rahman, Mohammed Ziaur Rahman, Mohammad Enayet Hossain, A. K. M. Monwarul Islam, Mir Jamal Uddin, Aye Moa, Alamgir Kabir, Timothy C. Tan, C. Raina MacIntyre, Ole Fröbert

**Affiliations:** ^1^ Biosecurity Program, The Kirby Institute, Faculty of Medicine and Health University of New South Wales Sydney New South Wales Australia; ^2^ Programme for Emerging Infections, Infectious Diseases Division, icddr,b Dhaka Bangladesh; ^3^ EMPHNET, Bangladesh Office Dhaka Bangladesh; ^4^ Virology Laboratory, Infectious Diseases Division, icddr,b Dhaka Bangladesh; ^5^ National Institute of Cardiovascular Diseases Dhaka (NICVD), Department of Cardiology Dhaka Bangladesh; ^6^ Biostatistics and Data Science Division, The George Institute for Global Health University of New South Wales Sydney New South Wales Australia; ^7^ Department of Cardiology, Blacktown Hospital University of Western Sydney Blacktown, Sydney New South Wales Australia; ^8^ School of Medical Sciences, Faculty of Medicine University of New South Wales Sydney New South Wales Australia; ^9^ Department of Cardiology, Westmead Hospital Sydney University Westmead, Sydney New South Wales Australia; ^10^ Faculty of Health, Department of Cardiology Örebro University Örebro Sweden; ^11^ Department of Clinical Medicine, Faculty of Health Aarhus University Aarhus Denmark; ^12^ Department of Clinical Pharmacology Aarhus University Hospital Arhus Denmark; ^13^ Steno Diabetes Center Aarhus Aarhus University Hospital Aarhus Denmark

## Abstract

**Background and Aims:**

COVID‐19 is an independent risk factor for cardiovascular disease. We investigated undiagnosed COVID‐19 and its effect on recurrent adverse cardiovascular events among patients with acute myocardial infarction (AMI).

**Methods:**

We enrolled patients with either ST‐segment elevation (STEMI) or non ST‐segment elevation myocardial infarction (NSTEMI) presenting at the National Institute of Cardiovascular Disease, Dhaka, from June 28 to August 11, 2020. Nasopharyngeal swabs were collected for SARS‐CoV‐2 testing by rRT‐PCR at enrolment. We followed all patients from admission until February 7, 2021, before the COVID‐19 vaccination in Bangladesh, to register clinical endpoints (all‐cause death, new AMI, heart failure, or new revascularization). Demographic information, cardiovascular risk factors, and clinical data were registered. Incidence rate (IR) per 100 person‐years follow‐up was calculated for clinical endpoints. Poisson regression was employed to estimate the incidence rate ratio (IRR) for SARS‐COV‐2 infection, adjusting for age.

**Results:**

We enrolled 280 patients with a mean age of 54.5 ( ± SD,11.8) years, and 78.6% were males. Of them, 12.9% had undiagnosed SARS‐CoV‐2 infection and were diagnosed with STEMI (*n* = 140, 50.0%) and NSTEMI (*n* = 140, 50.0%). We found that the IR per 100 person‐years of all cause death was 35.2, 95% CI: 25.6 to 48.5; recurrent AMI was 18.5, 95% CI: 12.1 to 28.2; heart failure was 6.7, 95% CI: 3.3 to 13.5; and revascularization was 23.5, 95% CI: 16.1 to 34.3. Patients with COVID‐19 had numerically higher IRRs for heart failure (2.40, 95% CI: 0.47 to 12.09, *p* = 0.290) and revascularization (1.11, 95% CI: 0.37 to 3.3, *p* = 0.853) compared to those without COVID‐19, though these differences were not statistically significant.

**Conclusion:**

This study provides updated data on undiagnosed cases among AMI patients during the first wave of the COVID‐19 pandemic. Our findings emphasize the need for further research to explore the impact of COVID‐19 on AMI patients in resource‐limited settings like Bangladesh.

## 1 Introduction

Observational studies have reported that individuals with SARS‐CoV‐2 infection suffered from impairment of myocardial and cardiac function [1, 2] and cardiovascular complications [3, 4, 5]. COVID‐19 has been considered an independent risk factor for cardiovascular diseases [5], and it may cause significant vascular pathology, cardiac injury, and acute and chronic cardiovascular complications [6].

During the initial phase of the COVID‐19 pandemic, most efforts were directed towards controlling and containing the virus, leaving limited resources for investigating its effects on major cardiovascular complications like acute myocardial infarction (AMI). Consequently, the pandemic and its associated control measures significantly restricted healthcare access, resulting in delayed and diminished care for cardiac emergencies [7].

Conducting studies on undiagnosed infections among patients with AMI presented significant challenges in resource‐limited settings such as Bangladesh. Despite these constraints, we investigated undiagnosed SARS‐CoV‐2 infections and their association with recurrent cardiovascular events during the first wave of the COVID‐19 pandemic. This study aimed to characterize and estimate the effect of COVID‐19 on the recurrent adverse cardiovascular outcomes among patients with AMI during the first wave of the COVID‐19 pandemic in Bangladesh.

## 2 Methods

We conducted a prospective longitudinal observational study at the National Institute of Cardiovascular Diseases (NICVD) Hospital in Dhaka, enrolling participants admitted for ST‐segment (STEMI) or non‐ST segment elevation myocardial infarction (NSTEMI) between June 28 and August 11, 2020. At the time of enrollment, nasopharyngeal swabs were taken to detect SARS‐CoV‐2 RNA using rRT‐PCR. The details of the study are published elsewhere [8]. After discharge, participants/family members were contacted to record target clinical endpoints including (i) all‐cause death, (ii) recurrent (new) AMI, (iii) heart failure, or (iv) new unplanned percutaneous coronary intervention (PCI) and/or stent thrombosis (revascularization). The follow‐up was scheduled via mobile phone calls: at Day 7, 1 month, and then every 3 months post‐enrollment until study completion. At each follow‐up, target endpoint data were collected and verified against medical records. Participants were asked to share medical records, such as prescription notes and hospital discharge summaries, via commonly used messaging apps over the internet. This was a feasible approach in Bangladesh, where mobile and internet penetration are high [9]. If multiple endpoints occurred during the follow‐up period, only the first event was included in the endpoint analysis. We also reported the national COVID‐19 detection rates throughout the study period. This follow‐up continued until the nationwide COVID‐19 vaccination campaign began in Bangladesh on February 7, 2021 [10], to avoid confounding by vaccination.

We used descriptive statistics to examine socio‐demographic information and medical history by type of AMI. Incidence rates (IR) per 100 person‐years of follow‐up were estimated for all clinical endpoints. Poisson regression was employed to estimate the incidence rate ratio (IRR) and their 95% confidence interval (CI) by SARS‐CoV‐2 infection status, adjusting for age. A *p*‐value of ≤ 0.05 was considered significant. Data were collected electronically and stored on a local server, and all analyses were conducted using Stata v.18 (StataCorp LP, College Station, TX, USA). Graphical illustration was created using R version 4.4.2, employing the ggplot2, dplyr, tidyr, and RColorBrewer packages.

The study received approval (protocol # PR‐20045) from the institutional review board of icddr,b on June 23, 2020. This review board is comprised of two separate committees known as the Research Review Committee and the Ethical Review Committee. All participants provided written informed consent before enrolling in the study.

## 3 Results

There were 280 patients with a mean age of 54.5 years (**±**11.8 SD), 220 (78.6%) of whom were male; there were 140 (50.0%) STEMI cases and 140 (50.0%) NSTEMI cases. The socio‐demographic and clinical characteristics are detailed in Table 1. Most patients resided in peri‐urban areas (48.6%), followed by urban (36.4%) and rural (15.0%) areas. Educational attainment varied, with nearly half (47.1%) having 1–5 years of school attendance. There were 36 (12.9%) patients who tested positive for SARS‐CoV‐2 at enrollment. The mean body mass index (BMI) was 23.4; 30.0% of patients were overweight (BMI > 25). Diabetes mellitus was present in 38.2% of patients, while smoking status was distributed among never smokers (40.0%) and current smokers (41.1%). Additionally, 16.8% of patients had hyperlipidemia, and 48.6% had hypertension. A history of AMI was recorded in 12.5% of patients. Previous PCI was significantly more common in the NSTEMI patients (7.1%) compared to the STEMI patients (2.1%, *p* = 0.047), and previous coronary artery bypass grafting (CABG) was only present among 3.6% of the NSTEMI patients.

**Table 1:** Baseline characteristics of AMI patients during the first wave of the COVID‐19 pandemic in Dhaka, Bangladesh.

	AMI patients *N* = 280	STEMI *N* = 140	NSTEMI *N* = 140	*p* value
Age, yr. (mean (± SD))	54.5 (11.8)	57.8 (11.2)	55.2 (12.3)	0.448
< 40	26 (9.3)	12 (8.6)	14 (10.0)	0.916
40–64	193 (68.9)	97 (69.3)	96 (68.6)	
≥ 65	61 (21.8)	31 (22.1)	30 (21.4)	
Male sex, no. (%)	220 (78.6)	112 (80.0)	108 (77.1)	0.560
Location of residence				
Peri‐urban	136 (48.6)	72 (51.4)	64 (45.7)	0.462
Urban	102 (36.4)	46 (32.9)	56 (40.0)	
Rural	42 (15.0)	22 (15.7)	20 (14.3)	
Education, years of school attendance				
None	67 (23.9)	37 (26.4)	30 (21.4)	0.588
1–5	132 (47.1)	65 (46.4)	67 (47.9)	
6–10	29 (10.4)	11 (7.9)	18 (12.9)	
11–12	27 (9.6)	15 (10.7)	12 (8.6)	
≥ 13	25 (8.9)	12 (8.6)	13 (9.3)	
Medical History				
SARS‐CoV‐2 positivity at enrolment	36 (12.9)	18 (12.9)	18 (12.9)	1.000
Body mass index (mean (± SD))^a^	23.4 (3.6)	23.3 (3.5)	23.5 (3.7)	0.802
Overweight (BMI > 25)	84 (30.0)	41 (29.3)	43 (30.7)	0.794
Diabetes mellitus, no. (%)	107 (38.2)	56 (40.0)	51 (36.4)	0.538
*Smoking status, no. (%)*				
Never smoked	112 (40.0)	55 (39.3)	57 (40.7)	0.898
Former smoker	53 (18.9)	28 (20.0)	25 (17.9)	
Current smoker	115 (41.1)	57 (40.7)	58 (41.4)	
Hyperlipidaemia, no. (%)	47 (16.8)	20 (14.3)	27 (19.3)	0.263
Hypertension, no. (%)	136 (48.6)	72 (51.4)	64 (45.7)	0.339
Previous myocardial infarction, no. (%)	35 (12.5)	13 (9.3)	22 (15.7)	0.104
Previous PCI, no. (%)	13 (4.6)	3 (2.1)	10 (7.1)	0.047*
Previous coronary artery by‐pass grafting, no. (%)	5 (1.8)	0	5 (3.6)	0.024*

^a^ Body mass index = kg/m^2^.

* Statistically significant.

### 3.1 Clinical Endpoints and Contemporary National COVID‐19 Positivity Rates

**Figure 1:**
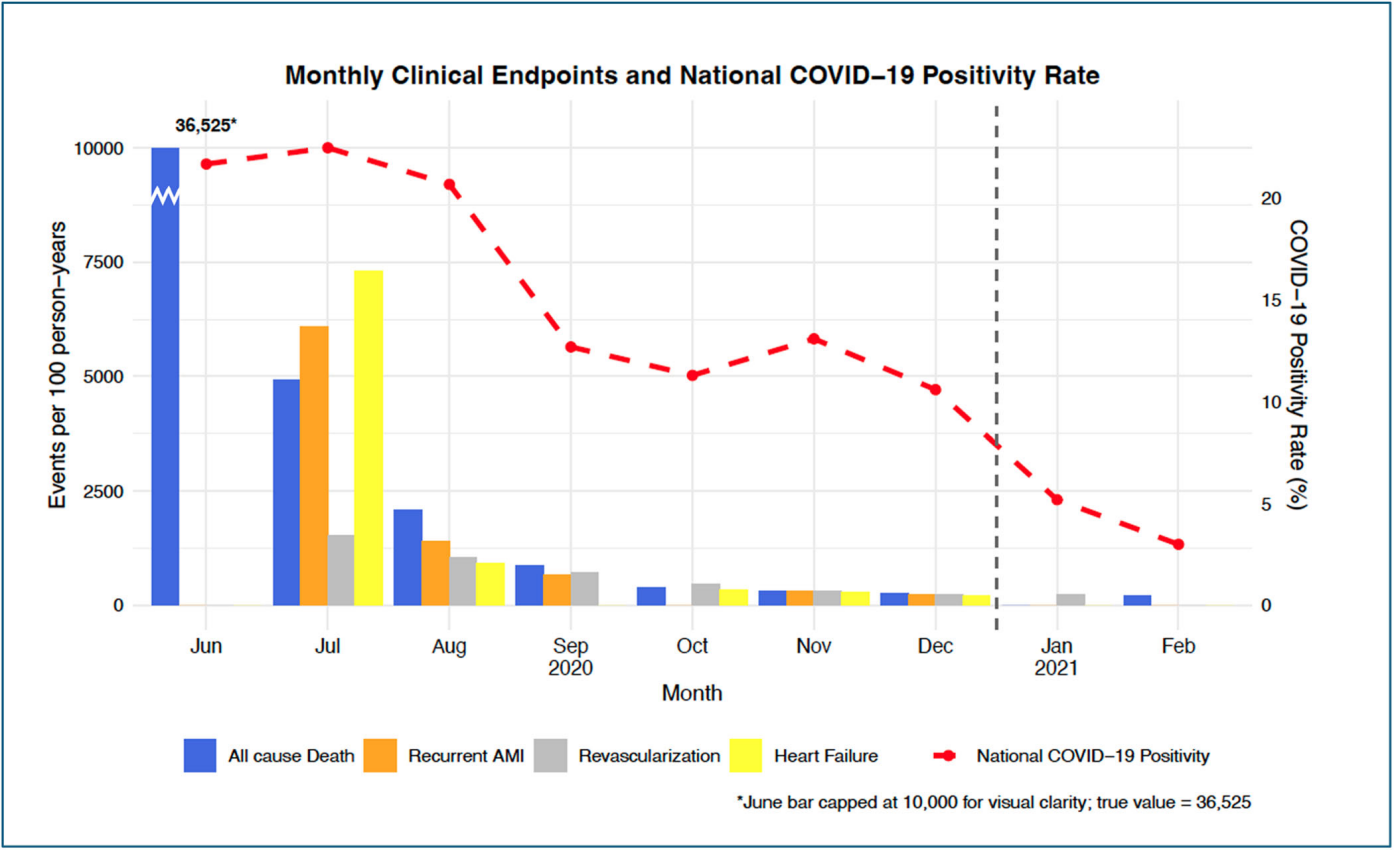
Distribution of clinical endpoints (events per 100 person‐years follow‐up) following AMI and National COVID‐19 positivity rates among patients with acute myocardial infarction during the first wave of the COVID‐19 pandemic in Bangladesh before the commencement of vaccination efforts (June 28, 2020–February 7, 2021).

During the recruitment period, 28 clinical events were documented. Over the entire study period, from June 28, 2020, to February 7, 2021, an additional 72 events were registered, bringing the total to 100 (35.7% of all participants recruited). These included 42 all‐cause deaths (15.0%), 22 cases of recurrent AMI (7.9%), eight heart failure events (2.9%), and 28 revascularizations (10.0%). Among the all‐cause deaths, 13 (31.0%) were in‐hospital deaths, including six (14.3%) following STEMI. In Figure 1, the monthly clinical endpoints per 100 person‐years follow‐up are illustrated. During the follow‐up period, national COVID‐19 positivity rates in Bangladesh fluctuated, peaking at 23% in July 2020 and gradually declining to 3% by February 2021 [11], marking the end of the first pandemic wave.

The IR per 100 person‐years follow‐up for all endpoints is detailed in Table 2. All cause deaths had an IR of 35.2 per 100 person‐years follow‐up (95% CI: 25.6 to 48.5), followed by that of revascularization, 23.5 per 100 person‐years follow‐up (95% CI: 16.1 to 34.3). The mean follow‐up period was 0.43 years (SD ± 0.21), equivalent to approximately 5 months.

**Table 2:** IR per 100 person‐years follow‐up among patients with AMI during the first wave of the COVID‐19 pandemic in Dhaka, Bangladesh.

Endpoints (*n* = 100)	Number of patients	IR per 100 person‐years follow‐up (95% Confidence intervals)
All cause death	42	35.2 (25.6 to 48.5)
Recurrent AMI	22	18.5 (12.1 to 28.2)
Heart failure	8	6.7 (3.3 to 13.5)
Revascularization	28	23.5 (16.1 to 34.3)

### 3.2 Association of SARS‐CoV‐2 Infection With Clinical Endpoints

In Table 3, the IRs of all‐cause death and heart failure among undiagnosed SARS‐CoV‐2 patients were 114 (95% CI: 102 to 128) per 100 person‐years follow‐up and 114 (95% CI: 107 to 120) per 100 person‐years follow‐up, respectively. During the study period, after adjusting for age, we observed a numerically higher IRR of 2.4 (95% CI: 0.47 to 12.09, *p* = 0.290) for heart failure among individuals with undiagnosed SARS‐CoV‐2 infection compared to those without the infection, although these differences were not statistically significant. Similarly, after adjusting for age, the IRR for revascularization was slightly higher at 1.11 (95% CI: 0.37 to 3.28, *p* = 0.853) among undiagnosed COVID‐19 cases compared to noninfected individuals, but this difference was also not statistically significant.

**Table 3:** Incidence of clinical endpoints and their association with SARS‐CoV‐2 infection among patients with AMI during the first wave of the COVID‐19 pandemic in Dhaka, Bangladesh.

Clinical endpoints	Adverse cardiovascular events		Unadjusted IRR (95% CI)	Adjusted* IRR (95% CI)	*p* value
	SARS‐CoV‐2 positive (*n* = 14)	SARS‐CoV‐2 negative (*n* = 86)			
	Events, (IR per 100 P‐Y, 95% CI)				
All cause death	5 (114, 102 to 128)	37 (102, 100 to 105)	0.92 (0.34 to 2.50)	0.96 (0.35 to 2.65)	0.941
Recurrent AMI	3 (100, 92 to 109)	19 (99, 97 to 102)	1.07 (0.32 to 3.65)	1.07 (0.31 to 3.64)	0.915
Heart failure	2 (114, 107 to 120)	6 (101, 97 to 105)	2.27 (0.46 to 11.10)	2.40 (0.47 to 12.09)	0.290
Revascularization	4 (95, 82 to 111)	24 (98, 95 to 100)	1.13 (0.39 to 3.34)	1.11 (0.37 to 3.28)	0.853

Abbreviations: CI, confidence intervals; IR, incidence rate; IRR, incidence rate ratio; P‐Y, person‐years.

* Adjusted for age.

## 4 Discussion

In our study, following an undiagnosed SARS‐CoV‐2 Infection, heart failure and all‐cause death occurred at high incidence both at 114 per 100 person‐years of follow‐up. While the initial 13‐week findings from our study [8] indicated numerically higher all‐cause mortality and revascularization rates among laboratory‐confirmed COVID‐19 patients, the extended follow‐up period provides further insights. Although our findings were not statistically significant, future work should explore the association between AMI and subsequent exacerbation of heart failure complications following an undiagnosed SARS‐CoV‐2 infection. Other studies demonstrated such an association [12, 13]. Available evidence indicates that myocardial injury is the most common adverse event following SARS‐CoV‐2 infection, caused by invasion of the cardiomyocytes by the virus and immune‐mediated mechanisms for cardiac injury [2, 14, 15, 16, 17]. These pathophysiological changes following COVID‐19 may increase the risk of recurrent cardiovascular events, including heart failure [17, 18, 19].

We also observed a high incidence of all‐cause deaths following undiagnosed SARS‐CoV‐2 infections in our study. A considerable proportion (14.3%) of these deaths occurred in‐hospital following a STEMI. This was substantially higher than the previously reported proportion of 3.2% among patients experiencing a STEMI in the same setting during the pre‐COVID‐19 period of 2017–2018 [20]. In other settings, studies from the pre‐COVID‐19 pandemic period reported in‐hospital mortality rates of 8.2% in India [21] and 6.2% in South Africa [22], which were lower than our findings. Our findings are consistent with early studies during the COVID‐19 pandemic from the USA, which indicated a rise in in‐hospital mortality following STEMI, with one study reporting an increase from 2.5% during 2019 to 4.5% in 2020, and in‐hospital mortality rates reported in another study to be as high as 15.2% [23, 24, 25]. Similarly, there have also been concurrent reports of an increase in in‐hospital mortality during the COVID‐19 pandemic in the United Kingdom, Spain, and Germany [26, 27, 28].

The high in‐hospital mortality observed in our study is likely multifactorial, with several contributing factors influencing these outcomes. Firstly, there may have been a delay between symptom onset and hospital presentation [29]. A pre‐COVID‐19 pandemic period study from Bangladesh reported that 48.9% of patients presented to the hospital after 12 h after AMI symptom onset [30]. Most of our respondents live in peri‐urban or rural areas, which may have contributed to the late presentation at the study hospital, which is located in an urban area. Additionally, access to healthcare was more challenging during the study period due to ongoing COVID‐19 pandemic control measures, including movement restrictions in Bangladesh [31, 32, 33]. Secondly, healthcare in Bangladesh is predominantly self‐financed by patients, which accounted for 74% of total healthcare expenditure according to a recent report [34]. The high cost of medical revascularization procedures, such as PCI or CABG, may pose a significant barrier for patients who require intervention. These factors likely contributed to the fact that none of our patients underwent revascularization procedures of primary PCI or CABG upon presenting to the study hospital. For AMI management, pharmacoinvasive reperfusion with fibrinolytics was indicated upon hospital arrival, alongside symptomatic pain treatment. Evidence suggests that delayed revascularization leads to higher in‐hospital deaths even in developed countries [35, 36]. These factors, combined with the high in‐hospital mortality rate among our study participants, highlight the critical need for timely and guideline‐recommended treatments for AMI.

Our study had several important limitations. Although COVID‐19 testing was conducted at enrolment, we could not assess subsequent infections after hospital discharge, which may have introduced effect modification. Reinfection by SARS‐CoV‐2 is known to carry an additional risk of cardiovascular events [37]. As the COVID‐19 pandemic progressed, infections and re‐infections, consistent with global trends, became more prevalent in Bangladesh [38, 39]. But testing for SARS‐CoV‐2 infections or reinfections after discharge from the study hospital was beyond the scope and available resources of the study. Additionally, the study was conducted in a single hospital in an urban location within a resource‐limited setting where guideline‐recommended treatments for STEMI/NSTEMI could not be routinely provided, which may have influenced the outcomes observed. Lastly, the study was not powered to ascertain the effects of COVID‐19 on recurrent cardiovascular events, therefore, caution should be exercised in interpreting the reported findings.

Despite the limitations, our study documented the incidence of adverse cardiovascular events, specifically all‐cause death and heart failure, among AMI patients following SARS‐CoV‐2 infection during the first wave of the COVID‐19 pandemic in Bangladesh. By establishing updated data on undiagnosed SARS‐CoV‐2 infections, our study highlights informed evidence for understanding the broader cardiovascular implications of the COVID‐19 pandemic in Bangladesh and other resource‐limited settings, underscoring the need for further research exploring the effects of SARS‐CoV‐2 infection in AMI patients.

## Author Contributions

**Zubair Akhtar:** conceptualization, investigation, funding acquisition, writing – original draft, methodology, writing – review and editing, formal analysis, project administration, data curation, software, visualization. **Fahmida Chowdhury:** conceptualization, investigation, supervision, resources, writing – review and editing. **Mohammad Abdul Aleem:** conceptualization, investigation, writing – review and editing; project administration. **Mahmudur Rahman:** conceptualization, methodology, resources, writing – review and editing, project administration. **Mustafizur Rahman:** validation, writing – review and editing, investigation. **Mohammed Ziaur Rahman:** validation, writing – review and editing, investigation. **Mohammad Enayet Hossain:** investigation, validation, writing – review and editing, data curation. **A. K. M. Monwarul Islam:** investigation, project administration, resources. **Mir Jamal Uddin:** investigation, project administration, resources. **Aye Moa:** writing – review and editing, methodology, supervision, formal analysis. **Alamgir Kabir:** formal analysis, software, validation, data curation, writing – review and editing, visualization. **Timothy C. Tan:** writing – review and editing, supervision, methodology. **C. Raina MacIntyre:** methodology, conceptualization, investigation, writing – review and editing, supervision, funding acquisition. **Ole Fröbert:** conceptualization, investigation, writing – review and editing, methodology, resources, supervision, funding acquisition.

## Conflicts of Interest

Ole Fröbert reports consultancy fees from GSK and speaker's fees from Pfizer and Sanofi. Timothy C. Tan reports consultancy fees from GSK and Bayer, as well as speakers's fees from GSK, Amgen, Novo Nordisk, Novartis, Boehringer Ingelheim and Sanofi. The remaining authors declare no conflicts of interest.

## Patient and Public Involvement

Patients and/or the public were not involved in the design, conduct, reporting, or dissemination plans of this study.

## Transparency Statement

The lead author Zubair Akhtar affirms that this manuscript is an honest, accurate, and transparent account of the study being reported; that no important aspects of the study have been omitted; and that any discrepancies from the study as planned (and, if relevant, registered) have been explained.

## Data Availability

Data generated during the study are subject to a data access policy of icddr,b and are available from icddrb's research administration on reasonable request through the corresponding author.
